# Gender differences in contrast-enhanced magnetic resonance imaging after acute myocardial infarction

**DOI:** 10.1186/1532-429X-14-S1-P24

**Published:** 2012-02-01

**Authors:** Birgit Langhans, Tareq Ibrahim, Albert Schömig, Stefan Martinoff, Martin Hadamitzky

**Affiliations:** 1 Deutsches Herzzentrum Muenchen, Munich, Germany

## Background

Besides different risk profiles for cardiovascular events in men and women, several studies reported gender differences in mortality after acute myocardial infarction (AMI). As infarct size has been shown to closely correlate with mortality, it is widely accepted as surrogate marker for clinical outcome. Currently, cardiovascular imaging studies covering the issue of gender differences are rare. As magnetic resonance scar characterization parameters are emerging as additional prognostic factors after acute myocardial infarction, we sought to evaluate gender differences in CMR infarct characteristics in patients after acute myocardial infarction.

## Methods

We prospectively analyzed patients (n=448) with AMI and primary angioplasty, who underwent contrast enhanced MRI imaging on a 1.5 T scanner in median 5 [3.8;5.6] days after the acute event. MRI scar size was measured 15 minutes after gadolinium injection. In addition presence and extent of MVO was assessed. A matched pair analysis was performed in order to exclude confounding by gender related co-morbidities and gender differences in established clinical risk factors.

## Results

Matching process according to clinical risk defined by GRACE score resulted in 93 mixed gender couples. Women were significantly older than men (64.4±11.9 vs. 60.5±12.3, p= 0.03) and presented with a significantly better ejection fraction before angioplasty (48.9±8.4 vs. 46.2±8.9, p=0.04). Infarct size did not differ significantly between women and men (13.5±10.7 vs. 15.1±11.8, p=0.32). Size of microvascular was significantly smaller in women than in men (0.48+-1.3 vs. 1.2+-3.0, p=0.03).

## Conclusions

Comparing scar characterization between women and men with similar risk profile revealed no gender differences in scar size. Size of MVO, however, was significantly smaller in women and might reflect better cardioprotective mechanisms in women. Whether these changes have prognostic implications has to be tested on a larger patient population.

## Funding

This trial was supported in part by the research grant from “Förderverein des Deutschen Herzzentrums”, Munich, Germany.

